# Development of a Quality Assurance Tool for Intensive Care Units in Lebanon During the COVID-19 Pandemic

**DOI:** 10.1093/intqhc/mzac034

**Published:** 2022-05-05

**Authors:** Märit Halmin, Ghada Abou Mourad, Adam Ghneim, Alissar Rady, Tim Baker, Johan von Schreeb

**Affiliations:** Department of Global Public Health, Karolinska Institutet, Stockholm, Sweden; The World Health Organization, Lebanon; Department of Global Public Health, Karolinska Institutet, Stockholm, Sweden; The World Health Organization, Lebanon; Department of Global Public Health, Karolinska Institutet, Stockholm, Sweden; Department of Global Public Health, Karolinska Institutet, Stockholm, Sweden

**Keywords:** COVID-19, Critical Care, Quality of Health Care, Checklist

## Abstract

**Background:**

During the COVID-19 pandemic, low- and middle-income countries have rapidly scaled up intensive care unit (ICU) capacities. Doing this without monitoring the quality of care pose risks to patient safety and may negatively affect patient outcomes. While monitoring quality of care is routine in high income countries, it is not systematically implemented in most low- and middle-income countries. In this resource scarce context there is a paucity of feasibly implementable tools to monitor quality of ICU care. Lebanon is an upper middle-income country that during the autumn and winter of 2020-21 has had increasing demands for ICU beds for COVID-19. The World Health Organisation has supported the Ministry of Public Health to increase ICU beds at public hospitals by 300% but no readily available tool to monitor the quality of ICU care was available. The aim of this study was to describe the process of rapidly developing and implementing a tool to monitor quality of ICU care at public hospitals in Lebanon.

**Methods:**

In the midst of the escalating pandemic, we applied a systematic approach to develop a realistically implementable quality assurance tool. We conducted a literature review, held expert meetings, and did a pilot study to select among identified quality indicators for ICU care that were feasible to collect during a one-hour ICU visit. In addition, a limited set of the identified indicators that were quantifiable were specifically selected for a scoring protocol to allow comparison over time as well as between ICUs.

**Results:**

A total of 44 quality indicators, that, using different methods, could be collected by an external person, were selected for the quality of care tool. Out of these 33 were included for scoring. When tested, the scores showed large difference between hospitals with low versus high resources, indicating considerable variation in quality of care.

**Conclusion:**

The proposed tool is a promising way to systematically assess and monitor quality of care in ICUs in the absence of more advanced and resource demanding systems. It is currently in use in Lebanon. The proposed tool may help identifying quality gaps to be targeted and can monitor progress. More studies to validate the tool is needed.

## Introduction

The COVID-19 pandemic has resulted in significant pressure on health systems globally, particularly in low- and middle-income countries (LMICs) where resources are limited [1]. Critical care is hospital-based care for the most severely sick patients and has been a key part of the response to the pandemic [2]. Critically ill patients in high income countries (HICs) are typically treated in intensive care units (ICUs) staffed with highly specialized health care workers and high-cost equipment and medication [3]. However, in most LMICs, ICU care is not available or is significantly constrained due to lack of resources.

Mortality outcomes following ICU care varied significantly between LMICs and HICs prior to the COVID-19 pandemic [4]. It is likely that these differences have increased further during the pandemic due to rapid increase of patients as well as urgent scale up of ICU beds without sufficiently added resources.

Quality of care is high on the global health agenda and is considered a crucial part of the work to reach universal health coverage (UHC) and Sustainable Development Goals 3 and 8 [5]. In HICs, assessment tools to measure and monitor hospital quality of care are part of regular routines and are well-studied [6-8]. By routinely collecting indicators of quality, gaps can be identified, and shortcomings addressed [9]. The use of quality assessment tools in hospital settings in LMICs is not well studied. However, it may be assumed that such tools could significantly contribute to improving mortality outcomes, with significantly greater effects compared to HICs [4].

Lebanon is an upper middle-income country where health care is mainly provided by a private, for-profit system. Before the compound crisis of the Lebanese liquidity crisis and the COVID-19 pandemic, 90% of hospitals beds were in the private sector (unpublished data from World Health Organisation Regional Office for Eastern Mediterranean). The public hospital system governed by the Ministry of Public Health (MOPH) is underfunded and public hospitals lack staff, equipment and medications [10].

Lebanon was relatively spared from COVID-19 during the spring of 2020. However, following the explosion in Beirut harbour in August, COVID-19 cases rapidly increased [10] triggering additional needs for COVID-19 hospital care and more ICU beds. At the end of August 2020, a total of 300 ICU beds, dedicated to COVID-19 care beds were available in Lebanon. Significant efforts were invested in scaling up the number of ICU beds and by April 1, 2021, a total of  1 176 ICU beds were available, out of which 90% were occupied [11]. In public hospitals, ICU beds almost tripled in less than eight months accounting for around 40% of total ICU beds available for COVID-19 care. However, there were significant concerns regarding quality care for critical ill COVID-19 patients, especially in public hospitals as they were severely understaffed and lacked systematic implementation of evidence-based protocols for ICU care (World Health Organization Lebanon, *Public hospitals assessment* 2020: Unpublished).

In this context, the World Health Organisation (WHO) in Lebanon focused its support to public hospitals to improve quality of care and upgrade their capacity through procurement of necessary equipment, coaching and hiring extra nursing staff. To ensure patient safety at the supported hospitals a tool to monitor quality of care in ICUs was urgently needed. However, a validated, easily implementable quality assessment tool for ICU care, that was applicable to the context, was neither readily available nor sufficiently described in the scientific literature. Despite this, it was essential to rapidly develop such a tool to be implemented while scaling up ICU beds in the midst of an escalating health emergency. The aim of this paper is to describe the ICU-care quality of care tool that was developed and the process of developing it. The tool is currently being used in 11 public COVID-19 ICUs in Lebanon.

**Methods**

In the rapidly evolving and urgent COVID-19 pandemic, we implemented a quality assurance project. Between September and November 2020 we created a tool to assess and document quality of care in the ICUs, using a systematic approach. We wanted the tool to be able to capture quality at a baseline assessment and assess changes in quality over time.
Ideally, a tool to monitor quality of care should be based on patient outcome data. However, in our setting such data was neither readily available as if so difficult to interpret due to significant case mix variations. For quality of care we sought inspiration for the Donobedian model that provides a framework to examine and evaluate quality of health care including ICU [28]. According to the model, information about quality of care can be drawn from three domains: structure, process, and outcomes. In our case we focused on the first two.

Besides from assessing quality of care we also wanted the tool to monitor changes following supportive interventions, such as the addition of extra nurses, introduction of high flow nasal cannula machines, implementation of new protocols, etc.

We defined an ICU according to the MOPH Lebanon accreditation standards classification and required the ICU to reach at least level II according to the international classification [3], which requires the ICU to have the possibility to provide invasive mechanical ventilation.

First, we conducted a literature review to identify potential indicators on structure and process for the checklist. We searched Pubmed using the key words “assessment”, “ICU”, “quality of care “, “minimal standards”, “low resource setting” and “quality indicators”. To assess the identified manuscripts we followed the PRISMA checklist [12]. From the literature, we extracted indicators that met the following criteria: 1) indicators that covered the domains of infrastructure, equipment and drugs, staffing, training and development, protocols and clinical management [13]; 2) indicators that were related to COVID-19 critical care, and 3) indicators that were considered feasible to collect. In this context, we assumed indicators were feasible to collect if they were  possible to collect within a one hour, or could be collected through observations, review of medical records, or by interviewing a responsible physician and nurse at each ICU [14]. We then reviewed the ICU quality of care indicators within the accreditation standards of the Lebanese MOPH [15], and identified and added further possible indicators.

Second, we sought the expert opinion of two experienced ICU physicians familiar with working in LMIC settings and three public health physicians well orientated in the healthcare system in Lebanon. The experts were asked to assess each indicator based on our selection criteria and to focus on creating a checklist that would be able to discriminate between ICU performing above or below minimum standards. Due to time limitations, the indicators were selected in one single face-to-face meeting with the five experts. Consensus among the five experts was defined a priori as prerequisite for an indicator to be included in the tool.

Finally, a pilot test was performed in one public hospital ICU to test the feasibility of collecting the selected indicators. To ensure the standardized use of the checklist, we created instructions for how to collect the data, including how to assess and categorize the parameters of each indicator. We stipulated that data collectors should be an experienced ICU physician or ICU nurse. We also performed a test where two data collectors filled in the checklist independently of each other in order to evaluate the concordance of the results. We added an additional specification to the instructions after this test.

Finally, we selected a group of indicators from the checklist to create a scoring protocol. The protocol was developed to provide numerical estimates of selected key indicators. This would allow monitor quality progress over time as well as enable comparison of results between ICUs. Indicators from the checklist were selected for the scoring protocol if they could be quantified and if they could be classified as either above or below minimum standards. The selection process was performed by the same expert opinion group. We decided on a simple protocol where each included indicator could generate zero or one point. If data was missing on any indicator, one point was subtracted from the total sum. Scoring results were displayed as both the sum of the score and the percentage of the maximum possible score.

To assess the checklist's ability to capture significant differences in quality of care between high versus low resourced ICUs, we conducted a test in one public hospital with low resources and two high-resourced university hospitals which had adopted internationally standardized protocols for ICU care. This was done under the assumptions that quality of care would vary between the hospitals [16].

This study documents a quality assurance project that is part of improving health care in Lebanon. As such there has been no formal ethical application submitted for writing up this study. No identifiable patient data was used and no individual has been exposed to any harms in this study.

**Results**

A total of 47 indicators were identified following the literature review and after revision of the national accreditation standards, one further indicator was added, for a total of 48 potential indicators. Three indicators were excluded following expert opinion and one indicator was excluded after the pilot, all due to doubts regarding the feasibility of collecting them (Figure 1). In the final checklist, 44 measurable indicators were included (see table 1).

*Insert Figure 1*

*Insert Table 1*

The comparison of results between two different collectors showed a high concordance; only one indicator differed in classification between the two collectors.

For scoring, 33 out of 44 indicators were selected by the expert opinion group. The indicators that were not selected were mainly descriptive. Although still valid for identifying possible support to an ICU, they were not able to classify as above or below any standard. The maximum score was 26, as 11 indicators were grouped (see table 2).

*Insert Table 2*

The scoring protocol was able to capture significant differences in quality of care. The public hospital ICU received a score of 13 points, or 52% of maximum total score. The first university hospital ICU scored 21 points (84%) and the second university hospital ICU scored 23 points (90%).

## Discussion

*Statement of principal findings*

The study has shown that it is possible to, in the midst of an escalating pandemic, during a short time and with limited resources, to develop and implement a checklist to monitor the quality of care in ICUs in Lebanon. Furthermore, scoring quality of care indicators seems to be a promising way to quantify the quality of care and compare between ICUs.

*Strengths and limitations*

The checklist was developed under significant time pressure. The main aim of the checklist was not research, but quality assurance. The strength of this manuscript is that it systematically documents the development and implementation of a quality assurance checklist in the midst of a pandemic with escalating ICU care needs. Without researched tools that assess quality of care, ethical issues arise, since the right to health care also includes the right to quality of care [17] and ensuring high-quality care is a mandatory step to reaching universal health coverage[5].

The rapid development of the checklist and the context has led to trade-offs and methodological considerations. A main one is linked to the selection of indicators and the checklist's ability to capture the quality of care. Quality of care is a dynamic concept including a range of aspects, some of which are difficult to measure [8, 18]. The selected indicators are not in any way comprehensive, but they capture different domains, with a main focus on respiratory care, and include many indicators that have already been adopted internationally. By conducting both a literature review and seeking expert opinion in the selection process, the validity of the checklist was justified to a certain degree [8]. However, more studies are needed to validate the checklist and its feasibility to be implemented in other resource-limited settings.

*Interpretation within the context of the wider literature*

Quality of care was originally described as including three domains: structure, process and outcome [18]. We expanded these domains to five, according to previous research on developing assessment tools in LMIC [13]. All five domains can be categorized under structure or process, as we actively chose to exclude outcome measures from the checklist. Outcome measures require data collection over time which can be burdensome [19]. Mortality is a main outcome of quality of care, but due to significant variations in COVID-19 patient mix at the ICUs, we found it impossible to use. Furthermore, the literature advocates that quality indicators should primarily focus on process and less on outcome measures [20]. The checklist should be seen as a complement to existing outcome indicators that are routinely collected, as it has the advantage of being easier to collect and less influenced by case-mix variation [21]. To improve quality of care, targeted interventions acting on the results of the assessment tool are needed. One such intervention that showed promising results was the introduction of a vital signs directed therapy protocol in an ICU in Tanzania [22]. Other interventions could emphasise the importance of the essential care of all patients with critical illness, both within and outside ICUs [23]. However, this requires political willingness and economic resources, as well as motivated staff.

Medical records are frequently used to extract quality of care indicators [24]. However, the quality and viability of collecting indicator data from records depends on reliable medical records and has been criticized as measuring what is documented rather than what is actually performed [25]. In this checklist, we complemented data from medical records with two more approaches: interviews [26] and direct observations. Using different approaches enables a more thorough picture of the reality in the ICU and increases the likelihood of generating robust results [27].

We tried to standardize data collection by including clear instructions with the checklist. Using explicit criteria to perform the assessment can increase the reliability of the process [28]. Our test showed a good concordance between two independent collectors and we further modified the instructions for better clarify following the test. Still, questions can be asked differently and observations can be disparate among data collectors, which risks producing measurement bias [29]. We therefore advocate to keep the number of data collectors as limited as possible to guarantee the internal validity of the results. All data collectors should, however, have expertise in ICU care.

Assigning scores to the indicators enables a more pedagogical communication of the results and facilitates comparison between different ICUs, between the same ICU over time, and before and after interventions in an ICU that are aimed at improving outcomes [30]. The indicators that were selected for scoring were those that could be dichotomized and could be defined as below or above minimum standards [31]. Unlike other assessment tools [26], we chose to only use dichotomized categorisation in order to simplify the assessment process. This may have reduced the tool's sensitivity to measure small differences between ICUs. However, it did facilitate the tool's ability to identify ICUs that were performing below standard.

There was no gold standard to compare to our scoring protocol's ability to capture differences in quality of care. However, we assumed that comparing hospitals with low versus high resources and hospitals that had or did not have standardized protocols before the COVID-19-pandemic could be used as a surrogate for levels of quality of care. Provided that our assumption is valid, a difference in 40 percentage points between hospitals with high versus low resources, indicates that our scoring protocol was able to capture a significant difference in quality of care.

*Implications for policy, practice and research*

The policy implication of the proposed tool is of limited value unless it is included in larger efforts to improve quality of care. It must be highlighted that our tool only provides quantitative and descriptive values and that documenting them will not automatically improve quality of care. However, given the extreme situation with escalating number of severely sick COVID-19 patients and dramatic scale up of ICU-bed we found it necessary to document quality systematically. To what extent this effort will improve quality remains to be seen as it will require efforts outside the mandate of WHO. Nevertheless, the quantifiable results document the situation and offers opportunities to numerically define. In upcoming papers, we will present the results of the scoring carried out. We hope that this paper will inspire colleagues in similar settings and hopefully serve as a basis for catalysing interventions for improvements. We also encourage colleagues in similar settings to write up their experiences of setting up systems to assess and monitor quality of care in ICUs in LMIC during the COVID-19 pandemic.

*Conclusion*

Our study shows that it is possible to develop and implement a tool to assess and monitor quality of care in ICUs in the midst of a pandemic, in a short time and with limited resources but more studies are needed to validate the tool. The tools checklist and scoring protocol is currently being used in 11 public ICUs in Lebanon.

Data sharing statement:

No additional data exist

Ethical approval statement:

This quality assurance project did not include data on individual participants nor did it disclose or reveal any individual medical information. The development and usage of the checklist is approved by ethical committee in Lebanon (USJ-2021-243).

Figure 1

Table 1: The 44 indicators selected for the quality of care tool categorized by domains

| **A Observations (at the moment of the visit)** | Subgroup |
|---|---|
| A01 Nurse/patient ratio | Staffing |
| A02 Person present at the central monitors | Staffing |
| A03 ICU patients intubated | Clinical management |
| A04 Patients on BiPAP or CPAP | Clinical management |
| A05 Patients on High Flow Nasal cannula | Clinical management |
| A06 Patients that fill discharge criteria | Clinical management |
| A07 No. of High Flow Nasal Cannula readily available in total | Clinical management |

| | |
|---|---|
| A08 No. of ventilators readily available in total | Clinical management |
| A09 All patients directly visible/or visible by camera | Infrastructure |
| A10 Clearly separated cleaned/dirty area | Infrastructure |
| A11 Complete Intubation equipment readily available | Equipment/Drugs |
| A12 Complete Intubation drugs readily available | Equipment/Drugs |
| A13 Readily available written protocols for intubation | Protocols |
| A14 Readily available written protocols for prone positioning | Protocols |
| A15 Any patient with asynchrony with ventilator | Clinical management |
| A16 Any patient with no peak pressure alarm set | Clinical management |
| A17 Intubated patients with head elevated 30 degrees | Clinical management |
| A18 No. of prone patients (intubated or awake) | Clinical management |
| A19 No. of unattended alarms during 10 minutes | Clinical management |
| **B Assessment of patient files/observational charts** | Subgroup |
| B01 No. of noted values of respiratory rate | Clinical management |
| B02 No. of noted values of minute volume or tidal volume | Clinical management |
| B03 No. of noted values of peak pressure | Clinical management |
| B04 No. of noted values of blood pressure | Clinical management |
| B05 Fluid balance last 24 hours | Clinical management |
| B06 Noted measures following last severely deranged value | Clinical management |
| B07 Arterial blood gas daily | Clinical management |
| B08 Measures undertaken following pathologic ABG results | Clinical management |
| B09 Electrolytes daily | Clinical management |
| B10 D-dimer at least 2 times per week | Clinical management |
| B11 Anticoagulation provided based on national guidelines | Clinical management |
| B12 Receiving proton pump inhibitors (PPI) | Clinical management |
| **C Questions to the present doctor/nurse/supervisor** | Subgroup |

| | |
|---|---|
| C01 Doctor rounds 2 times/day or more | Staffing |
| C02 How long did it take for doctor to arrive for last alarm | Staffing |
| C03 Time Specialist doctor present during yesterday | Staffing |
| C04 Nurse/patient ratio night shift (21.00-06.00) | Staffing |
| C05 Nurse/patient ratio weekend (Sat-Sun) | Staffing |
| C06 Emergency X-ray available for ICU patients | Infrastructure |
| C07 Echocardiography available within one day in ICU | Infrastructure |
| C08 Time from sampling to result of arterial blood gas | Infrastructure |
| C09 System for recording complications? | Training and development |
| C10 a) Thromboembolism diagnosed? b) If yes, how? | Clinical management |
| C11 a) Pneumothorax diagnosed? b) If yes, how? | Clinical management |
| C12 Morbidity and Mortality conference on regular basis | Training and development |
| C13 Responsible doctor Specialist with Critical Care Diploma | Staffing |

Table 2: Indicators selected for the scoring protocol, including points

| **Staffing (max 3p)** | |
|---|---|
| C14 Responsible doctor Specialist with Critical Care Diploma | 1p if yes, 0p if no |
| A01 Nurse/patient ratio ≥ 0,5<br>C04 Nurse/patient ratio night shift (21.00-06.00) ≥ 0,5<br>C05 Nurse/patient ratio weekend (Sat-Sun) ≥ 0,5 | 1p if all true,<br>0p if at least one false |
| C01 Doctor rounds 2 times/day or more | 1p if yes, 0p if no |
| **Infrastructure (max 5p)** | |
| A09 All patients directly visible/or visible by camera | 1p if yes, 0p if no |
| A10 Clearly separated cleaned/dirty area | 1p if yes, 0p if no |
| C06 Emergency X-ray available for ICU patients | 1p if yes, 0p if no |
| C07 Echocardiography available within one day in ICU | 1p if yes, 0p if no |
| C08 Time from sampling to result of arterial blood gas | 1p if ≤ 15 min, 0p if > 15 min |

| | |
|---|---|
| **Equipment/Drugs (max 2p)** | |
| A11 Complete Intubation equipment readily available | 1p if yes, 0p if no |
| A12 Complete Intubation drugs readily available | 1p if yes, 0p if no |
| **Clinical management (12p)** | |
| A17 Intubated patients with head elevated | 1p if ≥ 80%, 0p if < 80% |
| A18 No. of prone patients (intubated or awake) | 1p if 1 or more patients,<br>0p if 0 patients |
| B01 No. of noted values of respiratory rate > 12<br>B02 No. of noted values of minute volume or tidal volume > 6<br>B03 No. of noted values of peak pressure > 6<br>B04 No. of noted values of blood pressure > 12 | 1p if all true,<br>0p if at least one false |
| B05 Fluid Balance last 24 hours | 1p if yes, 0p if no |
| B07 Arterial blood gas daily | 1p if yes, 0p if no |
| B09 Electrolytes daily<br>B10 D-dimer at least 2 times per week | 1p if both true,<br>0p if at least one false |
| B11 Anticoagulation provided based on national guidelines<br>B12 Receiving proton pump inhibitors (PPI) | 1p if both true,<br>0p if at least one false |
| B06 Noted measures following last severely deranged value | 1p if yes, 0p if no |
| B08 Measures undertaken following pathologic ABG results | 1p if yes, 0p if no |
| A19 No. of unattended alarms during 10 minutes | 1p if no unattended alarm,<br>0p if unattended alarm exists |
| A15 Any patient with asynchrony with ventilator | 1p if no, 0p if yes |
| A16 Any patient with no peak pressure alarm | 1p if no, 0p if yes |
| **Training and development (max 2p)** | |
| C09 System for recording complications | 1p if yes, 0p if no |
| C13 Morbidity and Mortality conference on regular basis | 1p if yes, 0p if no |
| **Protocols (max 2p)** | |

| | |
|---|---|
| A13 Readily available written protocols for intubation | 1p if yes, 0p if no |
| A14 Readily available written protocols for prone positioning | 1p if yes, 0p if no |
| **Total max 26p** | |
| Missing data give zero points and one point is subtracted from the total | |
